# Bone marrow stromal cells from MDS and AML patients show increased adipogenic potential with reduced Delta-like-1 expression

**DOI:** 10.1038/s41598-021-85122-8

**Published:** 2021-03-15

**Authors:** Marie-Theresa Weickert, Judith S. Hecker, Michèle C. Buck, Christina Schreck, Jennifer Rivière, Matthias Schiemann, Katharina Schallmoser, Florian Bassermann, Dirk Strunk, Robert A. J. Oostendorp, Katharina S. Götze

**Affiliations:** 1 Department of Medicine III: Hematology and Oncology, School of Medicine, Klinikum Rechts Der Isar, Technical University of Munich, Ismaninger Str. 22, 81675 Munich, Germany; 2 Flow Cytometry Unit (CyTUM-MIH), Institute of Microbiology, Immunology, and Hygiene, Technical University of Munich, Munich, Germany; 3 Cord Injury and Tissue Regeneration Center Salzburg, Paracelsus Medical University, Salzburg, Austria; 4 Department for Blood Group Serology and Transfusion Medicine, Paracelsus Medical University, Salzburg, Austria; 5 German Cancer Consortium (DKTK), Heidelberg, Partner Site, Munich, Germany; 6 Experimental and Clinical Cell Therapy Institute, Paracelsus Medical University, Salzburg, Austria

**Keywords:** Mesenchymal stem cells, Cancer microenvironment, Myelodysplastic syndrome

## Abstract

Myelodysplastic syndromes (MDS) and acute myeloid leukemia (AML) are clonal hematopoietic stem cell disorders with a poor prognosis, especially for elderly patients. Increasing evidence suggests that alterations in the non-hematopoietic microenvironment (bone marrow niche) can contribute to or initiate malignant transformation and promote disease progression. One of the key components of the bone marrow (BM) niche are BM stromal cells (BMSC) that give rise to osteoblasts and adipocytes. It has been shown that the balance between these two cell types plays an important role in the regulation of hematopoiesis. However, data on the number of BMSC and the regulation of their differentiation balance in the context of hematopoietic malignancies is scarce. We established a stringent flow cytometric protocol for the prospective isolation of a CD73^+^ CD105^+^ CD271^+^ BMSC subpopulation from uncultivated cryopreserved BM of MDS and AML patients as well as age-matched healthy donors. BMSC from MDS and AML patients showed a strongly reduced frequency of CFU-F (colony forming unit-fibroblast). Moreover, we found an altered phenotype and reduced replating efficiency upon passaging of BMSC from MDS and AML samples. Expression analysis of genes involved in adipo- and osteogenic differentiation as well as Wnt- and Notch-signalling pathways showed significantly reduced levels of *DLK1*, an early adipogenic cell fate inhibitor in MDS and AML BMSC. Matching this observation, functional analysis showed significantly increased in vitro adipogenic differentiation potential in BMSC from MDS and AML patients. Overall, our data show BMSC with a reduced CFU-F capacity, and an altered molecular and functional profile from MDS and AML patients in culture, indicating an increased adipogenic lineage potential that is likely to provide a disease-promoting microenvironment.

## Introduction

Hematopoiesis is a tightly regulated process which is dependent on cues from the specific bone marrow (BM) microenvironment (niche) for regulation of self-renewal, differentiation and proliferation. The niche is composed of a variety of cells, among which bone marrow stromal cells (BMSC), a fraction of which are multipotent and form skeletal structures in vivo and thus also sometimes referred to as self-renewing osteoprogenitors^1^) or skeletal stem cells^2^. BMSC have been shown to play a key role in hematopoietic stem and progenitor cell maintenance. The term BMSC is used for both uncultured primary cells as well as cells growing in culture from fibroblast-like colonies (CFU-F). Although the term BMSC may mean different cell types for different investigators^3,4^, in this manuscript, we will use the terms primary BMSC or freshly-isolated BMSC for CFU-F-enriched cells isolated directly from cryopreserved BM samples by flow cytometry, and the term cultured BMSC for cells expanded from CFU-F-derived colonies. Freshly isolated primary BMSC were further classified as non-hematopoietic cells (CD45−/CD235a−) devoid of endothelial cell markers (CD31−). In addition, thus identified cells further expressing the nerve growth factor receptor CD271 have been shown to be highly enriched in CFU-F forming BMSC with multilineage potential in culture^5^. Cultured BMSC are expanded from these CFU-F and are further defined by plastic-adherence, a fibroblast-like morphology, the expression of a specific set of surface antigens and multilineage differentiation *in vitro*^6,7^. Increasing evidence suggests that BMSC are involved in development and progression of clonal myeloid disorders such as myelodysplastic syndromes (MDS) and acute myeloid leukemia (AML)^8,9^. However, studies of human primary BMSC have been hampered by isolation methods, as isolation by plastic-adherence has been shown to alter BMSC properties and surface marker expression, which may influence BMSC function in vitro^6,10–12^. In addition, plastic-adherence in culture does not yield a homogenous multipotent stromal cell population.

To study primary BMSC and interrogate their adipo-osteogenic differentiation potential, we developed a stringent flow cytometric sorting protocol for prospective isolation of rare CFU-F some of which give rise to multipotent BMSC from cryopreserved whole BM samples of healthy BM donors and newly diagnosed or currently untreated MDS and AML patients. Although this sorting procedure yields a population of cells with a homogeneous phenotype, the functional potential of these cells may still vary. Hence, we performed further functional analyses of this enriched population, through culture-expansion of CFU-F-derived BMSC and subsequent examination of their growth, differentiation, clonogenic capacity, and gene expression from BM samples of MDS and AML patients in comparison to age-matched healthy controls.

## Results

### The rare multipotent BMSC population can be isolated from cryo-conserved BM-MNC in MDS and AML, and healthy donor samples

**Figure 1:**
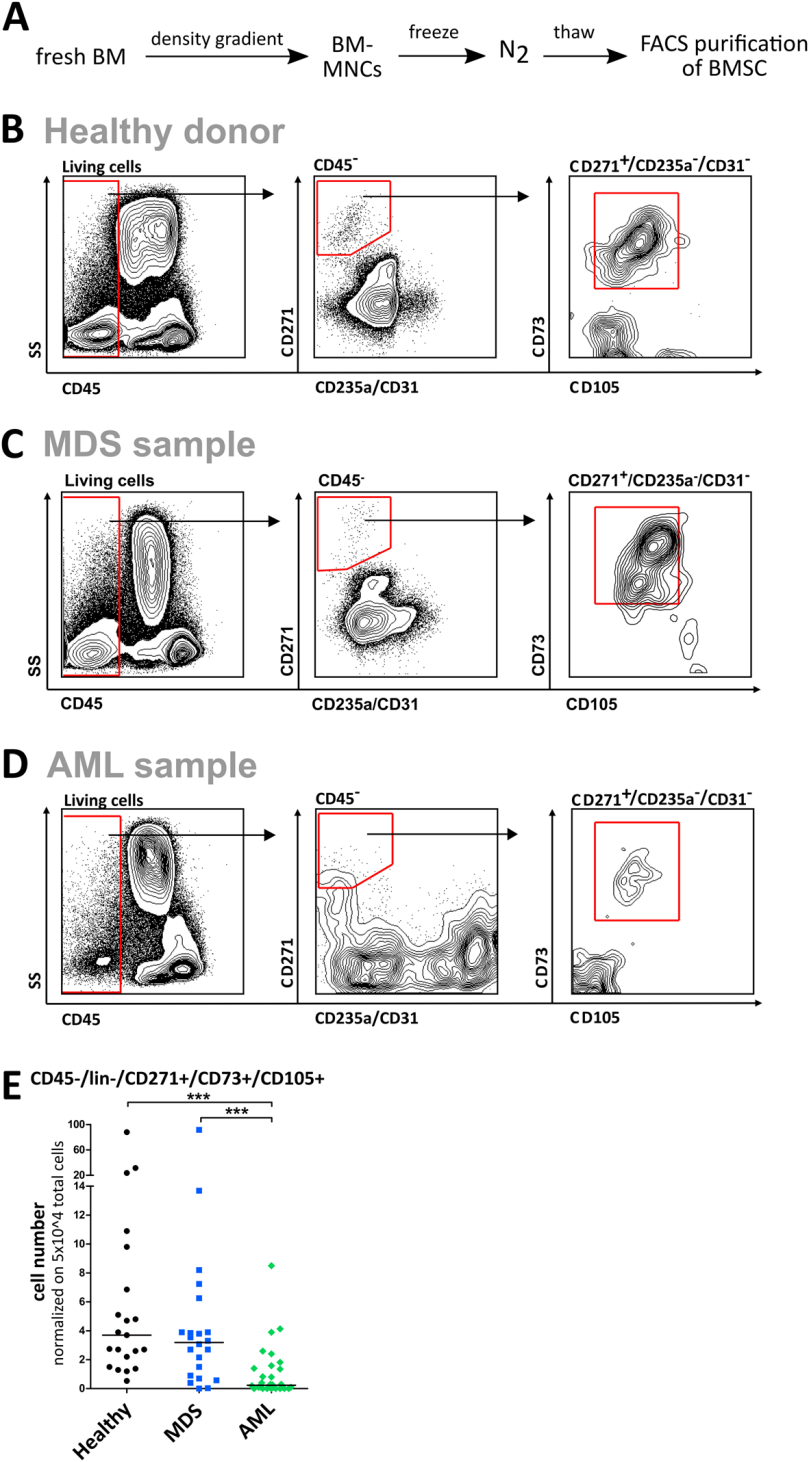
Isolation of the rare BMSC population from primary bone marrow samples. (**A**) Workflow: Mononuclear cells (MNCs) from fresh bone marrow (BM) aspirates of patients newly diagnosed with MDS or AML as well as healthy donors were obtained by density gradient centrifugation and stored in liquid nitrogen until use. For BMSC isolation, BM-MNCs were thawed and subsequently sorted by FACS. (**B–D**) Gating strategy for BMSC isolation. After forward/sideward scatter gating, doublet and dead cell exclusion (using propidium iodide staining), living cells were sorted on CD45^-^, lineage (CD235a/CD31)^-^, CD271^+^, CD73^+^ and CD105^+^ surface marker expression for purification of BMSCs. Representative FACS plots are shown for healthy donor (**B**), MDS (**C**), and AML (**D**) samples. (**E**) Uncultivated BM-MNC from healthy donors (n = 21), MDS (n = 22) and AML (n = 30) samples were analyzed on CD45−/CD31−/CD235a−/CD271+/CD73+/CD105+ expression by FACS, and statistical frequencies of the subpopulations were calculated. Event counts of the subpopulations were normalized on 5 × 10^4^ total event counts per sample. Medians are indicated in black.

To prospectively isolate the population of rare multipotent BMSC and study them without prior in vitro manipulation, we established a sorting protocol on the basis of previously published markers for BMSC^7^ (Fig. 1A). After exclusion of hematopoietic cells (CD45^+^), endothelial precursor cells (CD31 +), and erythrocyte precursor cells (CD235a^+^), cells were gated on the CD271^+^ population, which is currently thought to comprise most of the primary BMSC population^13^. When further narrowing down the population by expression of CD73 and CD105, which are part of the International Society for Cell Therapy minimum MSC criteria panel^7^, we found two subpopulations of CD271^+^ cells: the putative BMSC subpopulation, characterized by CD73 + /CD105 + expression (+/+ BMSC; Fig. 1 B-D, red boxes), and a population double-negative for both CD73 and CD105 expression (−/− BMSC). Using this protocol, primary BMSC were detected in frozen/thawed samples of healthy BM as well as BM aspirates from MDS and AML patients. However, the frequencies of +/+ BMSC were markedly lower in AML than in healthy and MDS BM samples (Fig. 1E). Cell numbers of BMSC recovered from diagnostic samples with this protocol were usually small, ranging from less than ten cells in the diseased samples to more than 10^4^ in healthy samples (median cell numbers: 3200 for healthy samples, 476 for MDS samples, and 70 for AML samples; normalized on 2 × 10^8^ input cells per sample and sort). This 7- to 45-fold reduction in MDS and AML samples, respectively may reflect the scarcity of the +/+ BMSC population in primary samples, but may also be a result of sensitivity to the cryo-conservation and thawing procedures. Taken together, our protocol allows for enumeration and isolation of viable putative BMSC from previously frozen samples.

### CFU-F represent a specific BMSC subset which is significantly reduced in malignant BM samples

**Figure 2:**
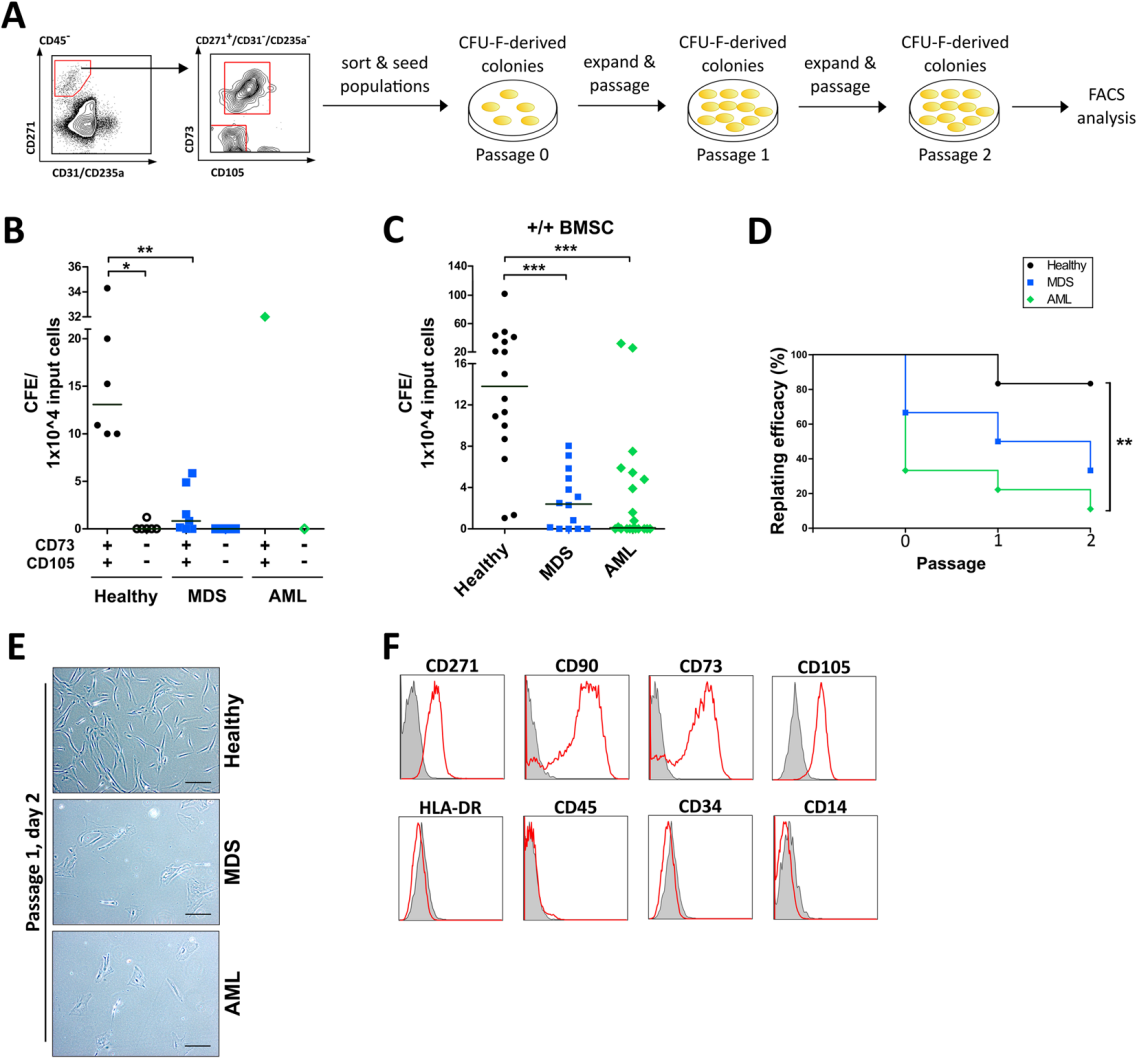
Formation of CFU-F-derived colonies is only found in a specific population subset and is strongly reduced in MDS and AML samples. (**A**) Experimental design. The CD45^−^/CD31^−^/CD235a^−^/CD271^+^/CD73^+^/CD105^+^ (+/+) and CD45−/CD31−/CD235a−/CD271^+^/CD73−/CD105− (−/−) subpopulations were simultaneously sorted from BM-MNC from healthy donors, MDS, and AML samples by FACS. Sorted subpopulations were seeded separately to assess their potential to form CFU-F-derived colonies, followed by re-analysis of their surface marker expression profile after two passages by FACS. (**B**) Colony-forming efficiency (CFE, normalized on 1 × 10^4^ input cells) in the +/+ and −/− subpopulations of healthy donors (n = 6), MDS (n = 7), and AML (n = 1) samples. Medians are indicated in black. (**C**) CFE (normalized on 1 × 10^4^ input cells) in the +/+ subpopulation of healthy donors (n = 16), MDS (n = 14), and AML (n = 20) samples. Medians are indicated in black. **(D**) Kaplan–Meier curve for replating efficiency (%) of +/+ sorted samples after initial seeding (0), passage 1, and passage 2. Healthy donors, n = 6; MDS, n = 6; AML, n = 9. (**E**) Representative light microscopy pictures showing the morphology of +/+ sorted cells from healthy donors, MDS, and AML samples at day 2 after passage 1. Cells were sorted, seeded and expanded at the same time and conditions. Scale bars = 100 µm. (**F**) Re-analysis of surface marker expression of +/+ sorted cells by FACS after passage 2. Representative histograms are shown for a healthy donor sample. Grey filled lines indicate IgG controls, red lines the respective antibodies.

Formation of replatable fibroblast-like colonies is a key cellular behaviour of multipotent BMSC^14^. To verify primary BMSC identity, both CD271^+^  +/+ BMSC expressing CD73 and CD105 and −/− BMSC were simultaneously sorted and seeded separately to assess their colony-forming efficiency (CFE) (Fig. 2A). The formation of fibroblast-like colonies was found exclusively in the +/+ BMSC population from healthy BM, MDS, and one exemplary AML sample (Fig. 2B) and not in the population expressing CD271 alone (−/− BMSC). In samples from healthy donors, sorting for +/+ BMSCs recovered 19 ± 3% (n = 6, SEM) of the CFU-F from unsorted BM cells with a 161 ± 37-fold enrichment in the +/+ BMSC subpopulation (Supplementary Fig. 1). Based on these findings, we focused solely on the +/+ BMSC subpopulation in the following experiments. When comparing the CFU-F frequencies of sorted primary +/+ BMSC, CFE were significantly reduced in MDS and AML compared to healthy BM (Fig. 2C). Analysis of sorted +/+ BMSC on initial formation of fibroblast-like colonies and subsequent survival over a period of two passages showed that only approximately 65% of all MDS and 30% of all AML samples contained CFU-F at all. Moreover, a notable decline in CFE for passaged BMSC from MDS was observed compared to healthy BM, which was further reduced in BMSC from AML BM (Fig. 2D). Furthermore, we observed morphological changes in culture-expanded +/+ BMSC in MDS and AML, that often appeared wider in diameter and flatter compared to parallel cultures of healthy donor BMSC (Fig. 2E). This is in line with previously published work, including our own, showing similar changes in cultured BMSC obtained by plastic adherence^15–17^. Cells expanded from CFU-F-derived colonies were re-analyzed by FACS to determine whether in vitro culture conditions altered their surface marker expression profile. We found that the BMSC cultured from sorted primary +/+ BMSC after two passaging steps remained positive for expression of CD271, CD90, CD73, CD105, and negative for CD45, HLA-DR, CD34, and CD14 expression, consistent with key BMSC criteria^7^ (Fig. 2F).

### The in vitro adipogenic differentiation potential is significantly increased in BMSC from MDS and AML samples

Since the adipo−/osteogenic balance of the niche is key to the regulation of hematopoiesis and this balance may be disturbed in the course of malignant transformation^18,19^, we analyzed the in vitro adipogenic and osteogenic differentiation potential of the sorted, culture-expanded +/+ BMSC from leukemic and healthy BM samples.

**Figure 3:**
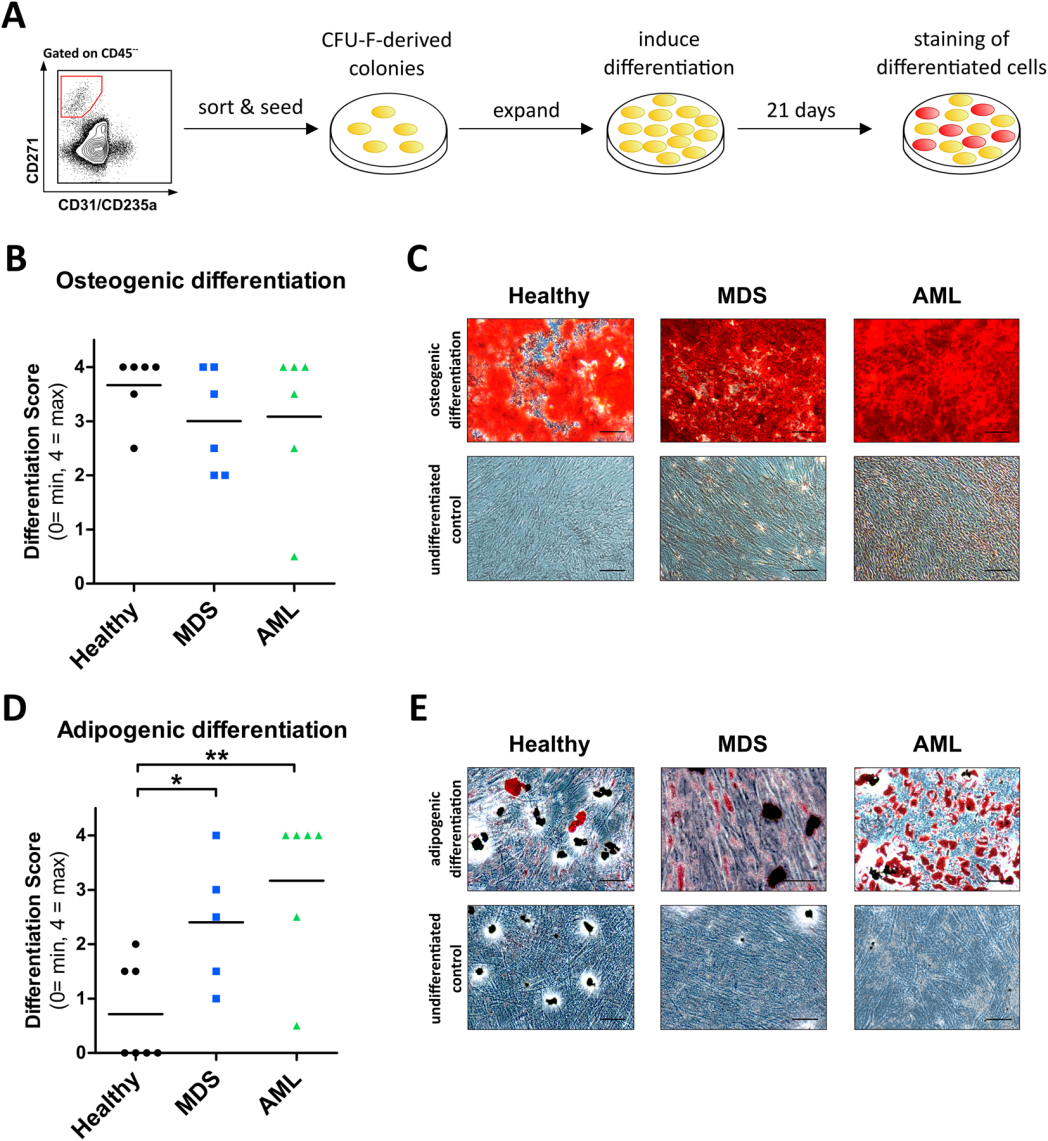
Significantly increased in vitro adipogenic differentiation potential in BMSCs from MDS and AML samples. (**A**) Experimental setup. CD45−/lin(CD31/CD235a)−/CD271^+^ sorted MSC from healthy donors, MDS, and AML samples were seeded and CFU-F-derived colonies were expanded until confluent. Osteogenic and adipogenic differentiation were induced in vitro for 21 days. Osteoblasts were detected by Alizarin Red staining of calcium deposits. Adipocytes were detected by Oil Red staining of fatty vacuoles. Stained samples were categorized according to a staining intensity score ranging from 0 (= no staining) up to 4 (= maximum staining). (**B**) Quantification of in vitro osteogenic differentiation potential of sorted BMSCs from healthy donors (n = 6), MDS (n = 6), and AML (n = 6) patient samples. Medians are indicated in black. (**C**) Representative images of Alizarin Red staining of osteogenic differentiated healthy donor, MDS, and AML samples (top row) and their respective undifferentiated controls (bottom row). Scale bars = 100 µm. (**D**) Quantification of in vitro adipogenic differentiation potential of sorted BMSCs from healthy donors (n = 7), MDS (n = 5), and AML (n = 6) patient samples. Medians are indicated in black. (**E**) Representative images of Oil Red staining of adipogenic differentiated healthy donor, MDS, and AML samples (top row) and their respective undifferentiated controls (bottom row). Scale bars = 100 µm.

For this purpose, CFU-F-derived colonies from sorted primary BM-derived +/+ BMSC from healthy donors, MDS, and AML samples were expanded and in vitro adipogenic and osteogenic differentiation was induced for 21 days (Fig. 3A). Calcium-deposits produced by osteoblasts were stained with Alizarin Red (Fig. 3 B,C) and adipocytes were detected by Oil Red staining of their fatty vacuoles (Fig. 3D,E). Adipogenic and osteogenic differentiation potential was quantified by the percentage of red pixels compared to undifferentiated control cultures and the subsequent calculation of a staining intensity score (0 = no staining, 4 = intensive staining, Supplementary Table 2). Surprisingly, a high osteogenic differentiation capacity was detected in BMSC from all sample types, with no significant differences in intensity levels observed between healthy, MDS, or AML samples (Fig. 3B). In contrast, a significantly increased adipogenic potential was found in BMSC from both MDS and AML samples compared to BMSC from healthy age-matched controls (Fig. 3D).

### Expression of the adipogenic inhibitor *Delta-like 1* (*DLK1*) is reduced in BMSC from MDS and AML samples

**Figure 4:**
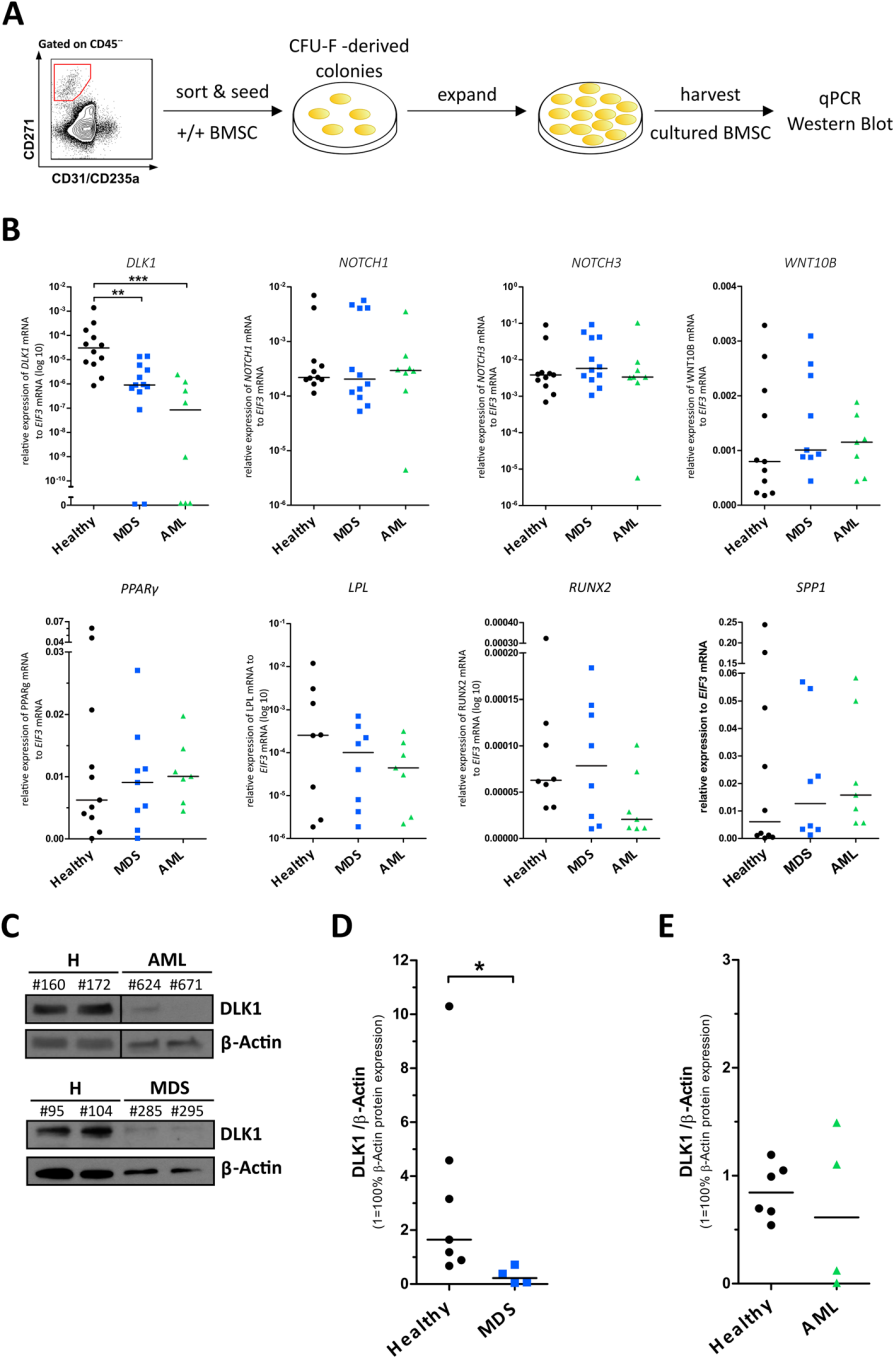
Expression of the adipogenic inhibitor Delta-like 1 (DLK1) is strongly reduced in MDS and AML BMSC. (**A**) Experimental setup: CD45−/lin(CD31/CD235a)−/CD271+/CD73+/CD105+ sorted +/+ MSC from healthy donors, AML and MDS samples were seeded and CFU-F-derived colonies were expanded and cultured BMSC harvested for gene and protein expression analysis. (**B**) Gene expression analysis by qRT-PCR of the orphan Notch/Delta/Serrate family-ligand DLK1, the Notch pathway receptors NOTCH1 and NOTCH3, the osteogenic cell fate stimulator WNT10B, the mature adipogenic differentiation markers PPARγ and LPL, and the mature osteogenic differentiation markers RUNX2 and SPP1 in cultured BMSCs from healthy donors (DLK1: n = 12, NOTCH1, NOTCH3, WNT10B, and PPARG: n = 11, each, LPL, and RUNX2: n = 8, each, SPP1: n = 10), MDS (DLK1: n = 13, NOTCH1 and NOTCH3: n = 12, each, PPARγ: n = 9, LPL, WNT10B, RUNX2, and SPP1: n = 8, each), and AML (DLK1, NOTCH1, NOTCH3, and WNT10B: n = 8, each, PPARγ, LPL, RUNX2, and SPP1: n = 7, each) samples. Data are shown as relative mRNA expression of the respective target gene to EIF3 mRNA expression using the ΔΔCt method with medians indicated in black. (**C–E**) Analysis of DLK1 protein expression in sorted BMSCs from healthy donors, MDS, and AML samples via immunoblotting. Exemplary immunoblots are shown in (**C**) with β-ACTIN as housekeeping control. Numbers indicate individual patient samples. H = healthy donors. Quantification of immunoblotting in (**D**,**E**) is shown as relative protein expression of DLK1 normalized on β-ACTIN expression, with 1 = 100% β-ACTIN expression. Medians are indicated in black. (**D**) Healthy donors n = 7, MDS n = 4. (**E**) Healthy donors n = 6, AML n = 4.

Since our results pointed to an altered lineage potential of BMSC from MDS or AML niches, we analyzed gene expression of several molecules involved in adipo- and osteogenic differentiation using quantitative RT-PCR of cultured +/+ BMSC from healthy and malignant samples (Fig. 4A). These experiments showed that the expression of late adipogenic (*PPARG* and *LPL*), as well as mature osteogenic (*RUNX1* and *SPP1*) markers did not differ between healthy and malignant samples (Fig. 4B). Interestingly, when looking at earlier markers in cell fate determination, such as the Notch inhibitory ligand *DLK1*, a known inhibitor of adipogenesis^20,21^, or *WNT10B*, a stimulator of osteoblasts^22^, we found significantly decreased expression o*f DLK1* in BMSC from MDS and AML samples, whereas expression levels of the Notch receptors *NOTCH1* and *NOTCH3* as well as *WNT10B* were not changed (Fig. 4B). To determine whether this finding was also displayed on a protein level, we analyzed DLK1 protein expression in sorted BMSC by immunoblotting (Fig. 4C-E). BMSC from all MDS samples and two out of four AML samples showed a strongly reduced DLK1 protein expression compared to healthy donor BMSC.

## Discussion

Significant knowledge has been generated through functional analysis of cultured BMSC isolated from whole BM samples using their ability to adhere to plastic surfaces like culture dishes^14^. This method for BMSC isolation is not ideal, as it requires a selection of adherent cells proliferating in mostly undefined culture media components, which results in a heterogeneous mixture of adhesive cells (including stromal cells, macrophages, endothelial cells, adipocytes or osteoprogenitors), and has no clearly defined starting population^12^. Recently, primary cells could be isolated directly from the BM which are enriched in CFU-F activity and allow culture expansion of BMSC after prospective isolation by FACS^13,23,24^.

We here describe a panel of surface markers enabling us to isolate primary BMSC from healthy donors, as well as MDS and AML BM samples which were previously cryopreserved. This panel combines the exclusion of hematopoietic and endothelial cells and the positive selection for expression of CD271 with positivity for both CD73 and CD105 (+/+ BMSC). This population is highly enriched for CFU-F and facilitates direct comparisons of +/+ BMSC from related diseases such as MDS and AML. Our observation that only this +/+ BMSC population comprises CFU-F which expand BMSC with adipocytic and osteogenic potential strongly suggests that the +/+ BMSC population contains rare multipotential BMSC.

Consistent with previous reports with plastic-adherence-^16^ as well as FACS-isolated BMSC^23^, we found strongly reduced CFE, diminished survival upon passaging, as well as morphological changes in +/+ BMSC from MDS and AML samples compared to healthy donors. These features suggest that BMSC from these diseases are subject to cellular stress. Indeed, this observation is in line with the increased inflammatory niche signature found in both primary CD271^+^ BMSC and cultured BMSC from MDS, as reported by Zambetti and colleagues^25^. These authors further showed that the transcriptome from primary BMSC isolated from BM samples and cultured BMSC displayed limited overlap, making direct comparisons of primary and culture-expanded BMSC difficult. Other studies also showed that cultured BMSC have a changed surface marker expression profile compared to freshly isolated, uncultivated cells^24,26^. For example, the distinctive CD271 marker was shown to be downregulated during prolonged in vitro culture of BMSC^27^.

In contrast to most other studies, we did not use fetal bovine serum (FBS) as a source for growth factors in the culture of CFU-F-derived BMSC, since the molecular composition of FBS is batch-dependent, and bears the risk of transmitting bovine pathogens and inducing xeno-immunization against bovine proteins^28,29^. Instead, we used pooled human platelet lysate (pHPL) according to a previously established protocol, that has lot-to-lot constant composition in crucial growth factors, promotes CFE, BMSC proliferation, and chromosomal stability better than culture-medium containing FCS, and bears no risk of xenogeneic immune responses^30,31^. In our study, re-analysis of FACS-isolated +/+ BMSC at passage 2 after expansion of CFU-F-derived colonies showed that these cells still robustly expressed CD271, CD73, CD105, and CD90, and lacked expression of other negative BMSC markers. This suggests that our prospectively isolated BMSC still maintained their surface marker profile after in vitro culture conditions and are therefore more likely to depict the properties of proper in vivo BMSC in experiments that need a larger number of cells, such as differentiation analyses.

The adipo−/osteogenic balance in the BM niche plays an important role in the regulation of hematopoiesis^18^. Nevertheless, the precise contributions of this balance to hematopoiesis and the development of malignancies have not been resolved in detail. There are several studies showing an inhibitory effect of adipocytes on healthy hematopoietic cells^19,32^. However, conflicting results were published on the adipo−/osteogenic differentiation potential in BMSC from MDS and AML patients suggesting mostly normal, but also elevated^33^, or reduced^34^ adipogenic potential of MDS BMSC (also reviewed in^35,36^). In our samples of BMSC cultured in pHPL, we observed a high osteogenic differentiation potential with unchanged osteogenic markers *WNT10B, RUNX2*, or *SPP1* for all of our prospectively isolated and CFU-F-derived BMSC in both healthy and malignant samples. Thus, our findings do not confirm decreased osteogenic potential observed in plastic-adherence selected malignant BMSC^15–17^. In line with our findings, Chen et al*.* observed expression of osteolineage markers in both prospectively isolated healthy and MDS BMSC using a nearly identical sorting strategy (CD45 − /7AAD − /CD235a − /CD31 − /CD271 + /CD105 +)^23^. This observation and our findings suggest that isolation of non-hematopoietic and non-endothelial CD271 + /CD105 + /CD73 + cells may select for an osteogenically primed population of primary BMSC that differs in its adipo-osteogenic potential compared to plastic-adherence-selected BMSC that likely comprise a more heterogeneous cell population. To distinguish in vitro and in vivo cellular differentiation potential of BMSCs derived from MDS and AML patients and determine skeletal stem cell potential, additional in vivo transplantation experiments could be helpful.

In our experiments, primary stringently prospectively isolated +/+ BMSC from BM samples of MDS and AML patients give rise to cultured BMSC with unchanged osteogenic potential, but with a significantly increased in vitro adipogenic differentiation potential. Our findings are in this respect similar to other reports of increased adipogenic potential in plastic-adherence-selected BMSC from MDS or AML patients^33,37,38^. It remains unclear whether different BMSC subpopulations with varying differentiation potential occur in the BM based on the respective BM sample source (such as femoral head vs. iliac crest aspirates) or their spatial localization within the BM niche. For instance, CXCL12-abundant reticular (CAR) cells with preferential potential for either adipo- or osteogenic differentiation (AdipoCAR and OsteoCAR) have been described in the murine system to be localized into different BM micro-niches^39^. However, similar adipo−/osteogenic biased subpopulations have not been characterized in human MSC thus far. To understand the altered differentiation potential between BMSC from MDS and AML samples, we therefore explored early and late differentiation markers. Surprisingly, although osteoblastic cell fate depending on *WNT10B* expression appeared not to be altered, we observed a strong reduction of the adipogenic cell fate inhibitor *DLK1* in MDS and AML BMSC samples on both gene and protein expression level. This could indicate that reduction of the inhibitory activity of DLK1 on adipogenic differentiation would drive adipogenesis in malignant BMSC^21,40–42^.

Besides its function as an adipocytic inhibitor, DLK1 was recently found to negatively regulate pro-inflammatory osteoclast and macrophage activation^43,44^. DLK1 thus acts as an anti-inflammatory mediator. Whereas deregulation of DLK1 expression has been described in hematopoietic cells from MDS patients^45^, the role of DLK1 expression in the stromal cell compartment has not been resolved. Our findings are in line with the observation that primary uncultivated MDS BMSC show an increased inflammatory signature^23,25^, suggesting that reduced DLK1 expression could contribute to this signature. A role for reduced DLK1 in inflammation noted in MDS and AML BMSC is further supported by findings showing that adipocytes induce a proinflammatory phenotype in residing AML blasts by secretion of molecules like TNFa and IL-1ß^46^. Thus, the downregulation of *DLK1* expression in MDS and AML BMSC observed in our study could contribute to pro-inflammatory signature of niche cells in myeloid malignancies. Intriguingly, obesity, which also induces inflammation and a plethora of metabolic changes^47^, has been noted as a significant clinical risk factor for the development of AML and MDS^48,49^. Although it is not clear from our data whether reduced DLK1 expression in the niche is triggered by AML cells themselves or is a precursor to the development of myeloid malignancy, the association between increased adipogenesis, inflammation and AML warrants further investigation^50^.

In summary, our findings indicate alterations in both numbers and CFU-F potential in prospectively isolated, primary BMSC from MDS and AML patient BM samples. Moreover, BMSC cultured from these CFU-F-derived colonies show increased in vitro adipogenic differentiation potential with reduced expression of the adipogenic cell fate inhibitor *DLK1* on gene and protein level in malignant samples. Thus, our findings point to a disruption of the adipo−/osteogenic balance within the BM stem cell niche in MDS and AML. Our results suggest an important role for deregulation of the *DLK1* gene in BM niche remodeling and disease progression in context of hematologic malignancies.

## Materials and methods

### Primary BM samples and Isolation of BM mononuclear cells

BM aspiration samples were collected from patients with newly diagnosed or currently untreated MDS and AML undergoing routine diagnostic evaluation after written informed consent. BM samples of healthy individuals without known hematologic disease were obtained from femoral heads after hip replacement surgery (Dr. Martin Nolde, SANA Klinik, München-Solln, Germany) after written informed consent and from collection bag filters used to filter donor bone marrow prior to further processing (Aktion Knochenmarkspende, Gauting, Germany). Femoral heads and collection bag filters were transported directly after collection procedures to our laboratory under sterile conditions. The use of all human samples was approved by the TUM Ethics Committee in accordance with the Declaration of Helsinki. Patient characteristics are shown in Supplementary Table 1.

BM was harvested by disintegrating and mincing the marrow of the femoral heads using bone-cutting forceps or by rinsing the collection bag filters with HF2 washing buffer (Hank’s balanced salt solution with 2% FBS and 10 µM HEPES buffer). BM fragments or washout were collected in tubes with PBS and BM cells were isolated mechanically by shaking the BM suspension. The cell suspension was then filtered with a 70 µm cell strainer and BM mononuclear cells (MNCs) were isolated by density gradient centrifugation through ficoll (density: 1.077 g/l). Cells were washed once in HF2 buffer and frozen in 10% DMSO and 90% FBS and stored in liquid nitrogen until use.

### Prospective isolation of uncultured BMSC from thawed BM mononuclear cells

Human mononuclear cells (MNC) were obtained from fresh BM aspirates and flushed BM donor filters via density gradient centrifugation (Biocoll, 1077 g/L, Merck, Darmstadt, Germany). MNCs were frozen in aliquots of max. 1e8 cells/vial in 90% FBS/10% DMSO and stored in liquid nitrogen until use. For flow cytometric isolation of primary BMSC, BM-MNCs were thawed, dead cells and cell debris removed by a second density gradient centrifugation, and unspecific binding was blocked by incubation in HF2 buffer (2% heat-inactivated FCS, 10 ml 1 M HEPES, 100 ml HBSS (100x), 10 ml penicillin–streptomycin (1e^4^ U/ml), 860 ml ddH_2_O). Cells were stained with the following surface marker panel: CD45-PE/Cy7 (clone HI30, eBioscience), CD31-eFluor 450 (clone WM-59, eBioscience), CD235a-eFluor 450 (clone HIR2, eBioscience), CD271-Alexa Fluor 647 (C40-1457, BD), CD73-PE (clone AD2, BD), CD105-FITC (clone SN6, Ancell), and CD90-PerCP/Cy5.5 (clone 5E10, eBioscience). In flow cytometric analyses and sorting experiments, after forward/sideward scatter gating, doublet and dead cell exclusion (using propidium iodide staining), the number of living cells was taken as total cells. Prospective BMSC were isolated by sorting living cells on CD45^-^, lineage (CD235a/CD31)^-^, CD271^+^, and further double positivity (+/+ BMSC) or double negativity (−/− BMSC) for CD73^+^ and CD105^+^ surface marker expression. Cell sorting was performed on a MoFlo Legacy (Beckmann Coulter, California, USA).

### Culture of prospectively isolated BMSC and CFU-F analysis

Cell culture of freshly isolated +/+ BMSC and −/− BMSC was performed as previously described^30,51^. Prospectively isolated BMSCs were seeded in BMSC medium, which consisted of low-glucose α-MEM (1 g glucose/L, M4526, Sigma-Aldrich), supplied with 2 mM L-glutamine, 10 U/L heparin (preservative-free, Merck, Darmstadt, Germany) and 20 U/ml penicillin–streptomycin. 10% (v/v) pooled human platelet lysate (prepared as described in^31^) was added freshly to the cell culture. Seeding density was on average 1000 cells/cm^2^ for healthy donor samples, or 2500 cells/cm^2^ for MDS and AML samples, respectively. Cells were propagated in a humidified incubator at 37 °C and 5% CO_2_. Two-thirds of the medium were changed twice a week.

Colony-forming efficiency (CFE) of fibroblast-like colonies was observed every second day for up to four weeks or until colonies grew confluent^52^. Colonies ≥ 25 cells were counted as being derived from CFU-F. −/− BMSC did not form fibroblast like colonies. CFU-F frequency was calculated by dividing the number of colonies observed by the number of input cells seeded, followed by normalization of colony number per 10^4^ input cells.

### Culture-expansion and flow cytometric analysis of CFU-F-derived BMSC

For expansion, CFU-F-derived BMSC were harvested using trypsin digestion and passaged in MSC medium, these multi-colony-derived BMSC were considered as passage 1 cells. For analysis of the expression of a set of commonly known surface marker molecules on cultured BMSC, cells of second passage were harvested and resuspended in HF2 buffer. Cells were aliquoted in separate vials and single-staining with the following antibodies was done for 30 min at 4 °C in the dark: CD45-FITC, CD90-PE, CD73-PE, CD105-FITC, CD271-Alexa Fluor 647, CD34-FITC, CD14-PE, HLA-DR-FITC. Additionally, single staining with respective IgG controls for antibodies and fluorochromes were performed at the same time. Cells were washed with HF2 buffer, resuspended in HF2 buffer + 1:1000 propidium iodide (1 mg/ml stock) and filtered directly before analysis on a CyAn ADP LxP8 flow cytometer (Beckman Coulter, California, USA) using the FlowJo software (version 7.6.5, FlowJo LLC., Oregon, USA).

### In vitro adipogenic and osteogenic differentiation of prospectively isolated BMSC

Cultured BMSC (passage 1 or 2) were expanded until confluent and in vitro differentiation was induced by replacing the normal BMSC medium with adipogenic (MSC medium supplemented with 1 µM dexamethasone, 60 µM indomethacin, 0,5 mM IBMX and 10 µM insulin) or osteogenic induction medium (BMSC medium supplemented with 1 nM dexamethasone, 0.1 mM L-ascorbic acid-2-phosphate, and at day 7 additionally with 10 mM β-glycerophosphate). Cells were cultured in induction medium for 21 days with complete medium changes thrice a week. For each sample, an additional cell culture without differentiation-inducing factors was set up and used as negative control.

For detection of adipocytes by Oil Red staining, cells were washed with PBS and fixed with formalin solution for 45 min at room temperature. Cells were washed with ddH_2_O and covered with Oil Red solution (0.5 g Oil Red O (Sigma) in 25 ml acetone and 25 ml 70% ethanol) for 3 min at room temperature. Oil red solution was removed, and cells were washed with doubly distilled H_2_O (ddH_2_O).

For detection of osteogenic differentiation potential, we stained for calcium deposits, a method which does not distinguish dystrophic calcification from matrix mineralization. For our purposes, we stained osteogenically induced BMSC using Alizarin Red. Cells were washed with PBS and fixed with 70% ethanol at − 20 °C for 1 h. Cells were rehydrated with ddH_2_O and covered with Alizarin Red solution (0,685 g Alizarin Red (Sigma) in 50 ml ddH_2_O, adjust to pH 4,1), followed by incubation for 10 min at room temperature with gentle rocking on a laboratory shaker. Alizarin Red solution was removed, and cells were washed carefully with PBS.

Stained samples were analyzed with a light microscope (Axiovert 25, Carl Zeiss Microscopy, Jena, Germany) and at least four random microscopic fields were digitized using AxioVision Software (Carl Zeiss Microscopy, Jena, Germany). For quantification of staining intensity, the differentiated areas were identified by applying a color threshold which allowed the identification of red pixels only. The percentage of red pixels (percentage of differentiated area) was normalized to a baseline calculated from undifferentiated, stained control samples. The normalized percentage of red pixels was converted to a differentiation score according to the scheme in Supplementary Table 2.

### Gene expression analysis of sorted and subsequently culture-expanded BMSC

Gene expression in cultured BMSC was measured by quantitative reverse transcription polymerase chain reaction (qRT-PCR), primers are listed in Supplementary Table 3 BMSC (first or second passage) were harvested using trypsin and total RNA was extracted using the RNeasy Mini Kit (Qiagen, Hilden, Germany) according to manufacturer’s instructions. Total RNA concentrations and ratios were determined by spectrophotometry (NanoDrop ND-1000, Thermo Scientific, Massachusetts, USA) and stored at − 80 °C. 1 µg RNA was transcribed to cDNA using the OmniScript RT Kit (Qiagen, Hilden, Germany) according to manufacturer’s instructions. qRT-PCR was performed with the Applied Biosystems Power SYBR Green PCR Master Mix (Thermo Scientific, Massachusetts, USA) using the StepOnePlus Real-Time PCR System (Thermo Scientific, Massachusetts, USA). For each patient sample, technical replicates were analyzed in triplicate. Relative mRNA expression was calculated by the ΔΔCt method and StepOnePlus Software (version 2.3).

### DLK1 protein expression in sorted BMSC

For analysis of DLK1 protein expression in BMSC, BM-MNC were sorted on CD45^−^/lin(CD31/CD235a)−/ CD271^+^ expression, seeded and expanded from CFU-F as described above. Cell lysis, SDS-PAGE and immunoblotting were performed as previously described^53^. Protein concentration was determined using the DC Protein Assay Kit II (Bio-Rad, California, USA). Protein absorption was measured at 750 nm using an ELx800 Universal Microplate Reader (BioTek Instruments Inc., Vermont, USA) and the BioTek Gen5 data analysis software (version 5.2). 40 µg total protein per sample were separated by SDS-PAGE in an electrophoresis chamber (30 min at 60 V, then 40 min at 120 V). Proteins were blotted onto a 0.45 µm PVDF membrane in a wet-transfer device with 1000 mA for 1 h at 4 °C. For detection of DLK1, the membrane was incubated overnight at 4 °C with anti-DLK primary antibody (ab119386, Abcam, Cambridge, UK) 1:1000 in 5% BSA in TBS-T. The membrane was washed and incubated with anti-rabbit IgG ECL HRP-linked secondary antibody (GE Healthcare, Little Chalfont, UK, 1:10,000 in 5% BSA in TBS-T). Antibody binding was visualized on light-sensitive screens (Kodak, NY, USA) using the SuperSignal West chemiluminescent substrate (Thermo Scientific, Massachusetts, USA). Signal intensity was analyzed using the ImageJ software (version 1.6.0). ß-ACTIN was detected as housekeeping control using the anti-ß-actin primary antibody (A5441, 1:5000, Sigma, Missouri, USA) and anti-mouse IgG ECL HRP-linked secondary antibody (GE Healthcare, Little Chalfont, UK, 1:10,000).

### Statistical analysis

Statistical analyses were performed by Mann–Whitney test, Wilcoxon signed rank test, Log-rank (Mantel-Cox) test and Gehan-Breslow-Wilcoxon test using GraphPad Prism software (version 5.01, GraphPad Inc, La Jolla, CA). *P* values are presented in the figures where a statistically significant difference was found: *, *P* < 0.05; **, *P* < 0.01; ***, *P* < 0.001.

## Supplementary Information

Supplementary Information.
