# Supplementary material for: Bone marrow stromal cells from MDS and AML patients show increased adipogenic potential with reduced Delta-like-1 expression
Source: Sci Rep. 2021 Mar 15;11:5944. doi: 10.1038/s41598-021-85122-8 (PMC7961144; doi:10.1038/s41598-021-85122-8)
Supplement: Supplementary file 1 — Supplementary Information. [file 41598_2021_85122_MOESM1_ESM.pdf]

Supplementary Files

Bone marrow stromal cells from MDS and AML patients show increased adipogenic potential with reduced Delta-like-1 expression

Marie-Theresa Weickert[1]*, Judith S. Hecker[1]*, Michèle C. Buck[1], Christina Schreck[1], Jennifer Rivière[1], Matthias Schiemann[2], Katharina Schallmoser[3], Florian Bassermann[1,4], Dirk Strunk[5], Robert A.J. Oostendorp[1#], Katharina S. Götze[1,4#]

[1]Technical University of Munich, School of Medicine, Klinikum rechts der Isar, Department of Medicine III: Hematology and Oncology, Munich, Germany.

[2]Technical University of Munich, Institute of Microbiology, Immunology, and Hygiene, Flow Cytometry Unit (CyTUM-MIH), Munich, Germany.

[3]Cord Injury & Tissue Regeneration Center Salzburg, Paracelsus Medical University, Salzburg, Austria; Department for Blood Group Serology and Transfusion Medicine, Paracelsus Medical University, Salzburg, Austria.

[4]German Cancer Consortium (DKTK), Heidelberg, Partner Site Munich, Germany.

[5]Experimental & Clinical Cell Therapy Institute, Paracelsus Medical University, Salzburg, Austria.

*Equal contribution, #Shared senior authorship.

Address correspondence to robert.oostendorp@tum.de or katharina.goetze@tum.de,

Ismaninger Str. 22, 81675 Munich, Germany

Word Count: Abstract 270; Introduction, Results and Discussion 2794

Figures: 4

Supplementary data: 3 Supplementary Tables, 1 Figure

Keywords: BMSC, MDS, AML, BM niche, adipogenesis, Dlk1

Supplementary Table 1. Sample and patient characteristics. Samples were collected as described in the Materials and Methods section. Abbreviations: H: healthy sample; MDS: myelodysplastic syndrome; AML: acute myeloid leukemia; m: male; f: female; n/a: not applicable or available; BM: bone marrow.

	Sample	Age	Gender	Sample source	Karyotype	Somatic mutations
Healthy donor samples	H 8/15	32	f	BM filter	n/a	n/a
	H 10/15	30	m	BM filter	n/a	n/a
	H 12/15	20	m	BM filter	n/a	n/a
	H 15/15	22	m	BM filter	n/a	n/a
	H 16/15	38	f	BM filter	n/a	n/a
	H 17/15	32	m	BM filter	n/a	n/a
	H 20/15	55	m	femoral head	n/a	n/a
	H32/15	48	m	BM filter	n/a	n/a
	H 34/15	42	m	BM filter	n/a	n/a
	H 37/15	37	m	BM filter	n/a	n/a
	H 38/15	42	m	BM filter	n/a	n/a
	H 43/15	29	m	BM filter	n/a	n/a
	H 45/15	26	m	BM filter	n/a	n/a
	H 48/15	78	m	femoral head	n/a	n/a
	H 68/15	47	m	BM filter	n/a	n/a
	H69/16	59	m	BM filter	n/a	n/a
	H 77/15	49	m	BM filter	n/a	n/a
	H 95/16	55	f	femoral head	n/a	n/a
	H 134/16	54	f	femoral head	n/a	n/a
	H 136/16	53	m	femoral head	n/a	n/a
	H 172/16	64	m	femoral head	n/a	n/a
	H 113/16	59	f	femoral head	n/a	n/a
	H 176/16	67	f	femoral head	n/a	n/a
	H 196/16	65	f	femoral head	n/a	n/a
	H 223/17	64	m	femoral head	n/a	n/a
	H 224/17	64	f	femoral head	n/a	n/a
	H 230/17	70	m	femoral head	n/a	n/a
	H 250/17	66	m	femoral head	n/a	n/a
	H 256/17	57	m	femoral head	n/a	n/a
	H 259/17	70	f	femoral head	n/a	n/a
	H 983/20	72	m	femoral head	n/a	n/a
	H 994/20	60	m	femoral head	n/a	n/a
	H 1036/20	68	f	femoral head	n/a	n/a

Supplementary Table 1. Sample and patient characteristics. Continued from previous page.

MDS samples

Sample	Age	Gender	Status at biopsy	Karyotype	Somatic mutations	WHO classification	IPSS	IPSS-R	BM fibrosis	Abnormalities besides hematological disease	Elevated fracture rate	BM fat cells (%)
MDS165/15	77	m	follow up	46, XY	ASXL1, SF3B1	MDS-RS-MLD	low (0)	low (3)	n/a	n/a	n/a	n/a
MDS 169/15	74	m	initial diagnosis	46, XY	TET2	CMML-I	int-I (0.5)	intermediate (3.5)	n/a	n/a	n/a	n/a
MDS 173/15	77	f	progressing disease	46, XX	SF3B1	MDS-RS-MLD	int-I (0.5)	intermediate (4)	n/a	n/a	n/a	n/a
MDS 180/15	70	m	progressing disease	45, XY, -7	Kit-D816V	MDS*	-	-	no	no	no	30%
MDS 182/15	66	f	progressing disease	complex	TP53	MDS*	-	-	no	bone rarefaction (low grade)	no	40%
MDS 208/15	75	m	progressing disease	46, XY	RUNX1, TET2, ASXL1	MDS-MLD	int-I (0.5)	intermediate (4.5)	n/a	n/a	n/a	n/a
MDS 210/15	70	m	progressing disease	47, XY, +8	ASXL1, RUNX1	MDS*	-	-	no	no	no	n/a
MDS 215/15	61	f	progressing disease	47, XX, +13	TET2, SRSF2, ASXL1, RUNX1	MDS/MPN overlap	int-II (2)	high (6)	no	bone rarefaction (low grade)	no	<10%
MDS 216/15	87	m	progressing disease	complex	ASXL1, SF3B1	MDS-MLD	int-I (0.5)	intermediate (4.5)	n/a	n/a	n/a	n/a
MDS 222/15	84	m	progressing disease	46, XY	ETV6, ASXL1, EZH2	MDS-MLD	int-I (0.5)	intermediate (4)	no	no	no	25%
MDS 227/15	68	f	progressing disease	46, XX	TET2, SF3B1	MDS-EB1	int-I (0.5)	low (3)	no	no	no	10%
MDS 228/15	74	f	initial diagnosis	46, XX	TET2, SRSF2	CMML-I	int-I (0.5)	low (3)	n/a	n/a	n/a	n/a
MDS 241/16	75	f	progressing disease	46, XX	SF3B1	MDS-RS-SLD	low (0)	low risk (2)	no	no	no	n/a
MDS245/15	40	m	initial diagnosis	47, XY, +8	ASXL1, KMT2A	MDS*	-	-	no	no	no	<10%
MDS 261/16	79	m	progressing disease	46,XY	SF3B1, ASXL1	MDS-RS-MLD	low (0)	low (3.5)	n/a	n/a	n/a	n/a
MDS 262/16	73	m	initial diagnosis	46, XY	no known mutations	MDS-MLD	low (0)	low risk (2)	no	no	no	<10%
MDS 263/16	81	f	initial diagnosis	46, XX	no known mutations	MDS-MLD	low (0)	low (3)	no	bone rarefaction (low grade)	no	30%
MDS 285/16	50	m	initial diagnosis	46, XY	SF3B1	MDS-RS-MLD	low (0)	low risk (2)	n/a	n/a	n/a	n/a
MDS 295/16	82	f	initial diagnosis	46, XX	no known mutations	MDS-MLD	low (0)	low risk (1.5)	no	no	no	80%
MDS 303/16	70	m	initial diagnosis	46, XY	SF3B1	MDS-MLD	low (0)	low risk (1.5)	no	no	no	50%

* later: progression to sAML

AML samples

Sample	Age	Gender	Status at biopsy	Karyotype	Somatic mutations	WHO classification	2017 ELN risk stratification	CD34 blasts	BM fibrosis	Abnormalities besides hematological disease	Elevated fracture rate	BM fat cells (%)
AML (M 245/15)	41	w	initial diagnosis	47, XX, +8	no known mutations	AML, not otherwise specified (NOS)	intermediate	n/a	no	no	no	50-60%
AML (M 176/15)	77	m	initial diagnosis	n/a	n/a	unkown	unkown	n/a	no	no	no	40%
AML 624/14	75	m	initial diagnosis	46, XY	no known mutations	AML with myelodysplasie-related changes (MRC)	intermediate	positive	grade 1	no	no	<10%
AML 671/15	59	f	initial diagnosis	complex	CBFB/MYH11 gene fusion	AML with myelodysplasie-related changes (MRC)	adverse	positive	no	bone rarefaction (low grade)	no	20%
AML 675/15	59	f	initial diagnosis	46, XX	NPM1	AML with myelodysplasie-related changes (MRC)	favorable	positive	no	bone rarefaction (low grade)	no	20%
AML 677/15	62	m	initial diagnosis	47, XY, +8	RUNX1	AML with recurrent genetic aberrations	adverse	positive	no	no	no	10%
AML 681/15	56	m	initial diagnosis	complex	no known mutations	AML with myelodysplasie-related changes (MRC)	adverse	positive	no	no	no	10%
AML 688/15	84	f	initial diagnosis	48, XY, +11, +13	no known mutations	AML, not otherwise specified (NOS)	intermediate	positive	no	no	no	10%
AML 690/15	63	f	initial diagnosis	46, XX	no known mutations	AML, not otherwise specified (NOS)	intermediate	positive	grade 1	no	no	5%
AML 701/15	62	m	initial diagnosis	47, XY, +8	no known mutations	AML, not otherwise specified (NOS)	intermediate	positive	no	no	no	5%
AML 722/15	59	f	initial diagnosis	46, XX, inv(16)(p13.1q22)	CBFB/MYH11 gene fusion	AML with recurrent genetic aberrations	favorable	n/a	no	bone rarefaction (low grade)	no	20%
AML 747/15	21	m	initial diagnosis	46,XY,t(8;21)(q22;q22)	RUNX1/RUNX1T1 fusion transcript	AML with recurrent genetic aberrations	favorable	n/a	n/a	n/a	n/a	n/a
AML 766/15	59	m	initial diagnosis	46, XY	no known mutations	AML, not otherwise specified (NOS)	intermediate	negative	no	no	no	<10%
AML 772/15	46	m	initial diagnosis	complex	TP53	AML with myelodysplasie-related changes (MRC)	adverse	positive	grade 1	no	no	50%
AML 781/15	73	f	initial diagnosis	complex	no known mutations	AML with myelodysplasie-related changes (MRC)	adverse	positive	no	bone rarefaction (low grade)	no	30%
AML 808/15	84	m	initial diagnosis	46, XY	no known mutations	AML, not otherwise specified (NOS)	intermediate	n/a	n/a	n/a	n/a	n/a
AML 814/16	36	f	initial diagnosis	46, XX	no known mutations	AML, not otherwise specified (NOS)	intermediate	negative	n/a	n/a	n/a	n/a
AML 842/16		m	initial diagnosis	n/a	n/a	unkown	unkown	n/a	n/a	n/a	n/a	n/a
AML 856/16	53	f	initial diagnosis	46, XX	RUNX1, SF3B1	AML with recurrent genetic aberrations	adverse	positive	no	no	no	85%
AML 858/16	61	m	initial diagnosis	47, XY, +11	no known mutations	AML, not otherwise specified (NOS)	intermediate	positive	no	no	no	40%
AML 860/16	60	m	initial diagnosis	47, XY, +8	no known mutations	AML, not otherwise specified (NOS)	intermediate	negative	grade 1	no	no	80%
AML 880/16	52	m	initial diagnosis	46, XY	ASXL1, CBL, KRAS, U2AF1	AML with myelodysplasie-related changes (MRC)	adverse	negative	n/a	n/a	n/a	n/a

Supplementary Table 2: Differentiation score.

Score	Osteogenic	Adipogenic
4	≥ 80 %	> 24 %
3.5	≥ 70 % - <80 %	≥ 12 % - <24 %
3	≥ 60 % - <70 %	≥ 6 % - <12 %
2.5	≥ 50 % - <60 %	≥ 4.5 % - <6 %
2	≥ 40 % - <50 %	≥ 3 % - <4.5 %
1.5	≥ 30 % - <40 %	≥ 1.5 % - <3 %
1	≥ 20 % - <30 %	≥ 1 % - <1.5 %
0.5	≥ 10 % - <20 %	≥ 0.5 % - <1 %
0	0 % - <10 %	0 % - <0.5 %

Supplementary Table 3: qRT-PCR primer sequences and GenBank accession numbers.

Gene	GenBank accession no.	Primer	Sequence
DLK1	NM 003836.7	forward	GACGGGGAGCTCTGTGATAG
		reverse	GGGGCACAGGAGCATTCATA
NOTCH 1	NM 017617.5	forward	TGAATGGCGGGAAGTGTGAAG
		reverse	CACAGCTGCAGGCATAGTC
NOTCH 3	NM 000435.3	forward	TTACTACCGAGCCGATCACC
		reverse	CAGAGGAGCTACTGCGTTCC
EIF3	NM 013234.4	forward	TGTCGGACAGCCAGCTAAAG
		reverse	CCATGATGCTGGACACACTG
PPARg	NM 138712.5	forward	GCTGGCCTCCTTGATGAATAA
		reverse	ACTCAAACTTGGGCTCCATAAA
LPL	NM 000237.3	forward	TTGGGATACAGCCTTGGAGC
		reverse	CAGGAGAAAGACGACTCGGG
RUNX2	NM 001015051.4	forward	CAACTTCCTGTGCTCGGTGC
		reverse	CCCGCCATGACAGTAACCAC
SPP1	NM 001040058.2	forward	GAAGTTTCGCAGACCTGACAT
		reverse	GTATGCACCATTCAACTCCTCG
WNT10B	NM 003394.4	forward	AGAGATCACCCACTCCTATGT
		reverse	TCTCTACCACTGTCTCCCATTA

Supplementary Figure 1

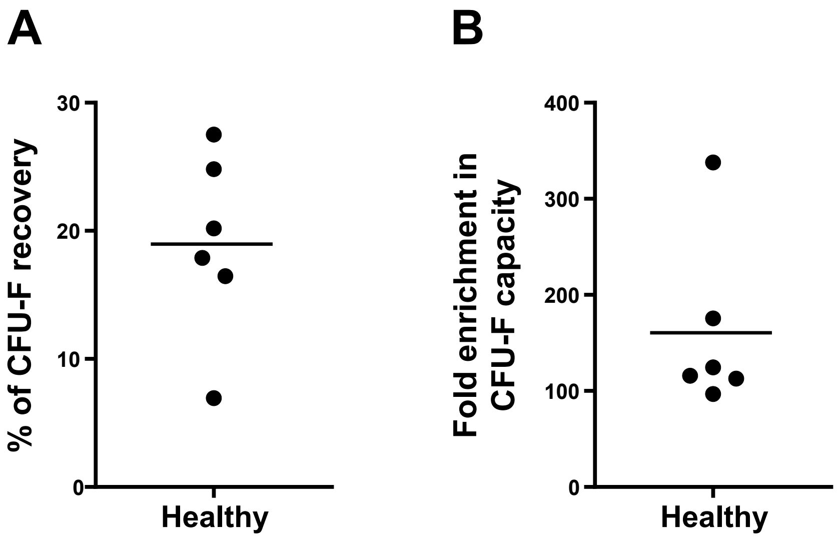

Supplementary Figure 1: Percentage of CFU-F recovery (**A**) and fold enrichment in CFU-F (**B**) from sorted CD271+/CD73+/CD105+ cells compared to unsorted BM MNC. Mean: indicated as black bar.
